# Sclerosing Angiomatoid Nodular Transformation of the Spleen: Analysis of Clinical and Pathological Features in Five Cases

**DOI:** 10.3389/fsurg.2020.609284

**Published:** 2021-02-01

**Authors:** Huijiang Shao, Baochun Lu, Zhihong Shen, Fang Liu

**Affiliations:** Department of Hepatobiliary and Pancreatic Surgery, Department of Pathology, Shaoxing People's Hospital, Shaoxing Hospital of Zhejiang University, Shaoxing, China

**Keywords:** spleen, splenectomy, sclerosing, angiomatoid nodular, transformation

## Abstract

**Objective:** We aimed to summarize the clinical and pathological features of sclerosing angiomatoid nodular transformation (SANT) in spleen among five cases.

**Methods:** Five cases (male: 3; female: 2; mean age: 47.6 years) with SANT confirmed by pathological analysis between July 2010 and November 2019 in our hospital were included in this study. The clinical, imaging, and pathological data were analyzed retrospectively.

**Results:** Three patients presented with mild abdominal pain or discomfort, while the other two were symptom free. Two patients received ultrasonography (US), and all patients underwent a computerized tomography (CT) scan in our hospital. The typical “spoke wheel” pattern was seen in two cases, and central calcification was detected in one case on the CT scans. All patients indicated peripheral enhancement around the SANT lesion during the arterial phase. Open or laparoscopic splenectomy was performed for treatment. No patient showed recurrence in the follow-up. The pathological characteristics of our cases were in line with those of previous literatures.

**Conclusions:** Peripheral enhancement around the SANT lesion during the arterial phase should be taken into consideration for the diagnosis of SANT as an imaging sign on CT scans. Special attention should be paid to the splenic integrality during the laparoscopic approach, due to the probability of malignancy and the fragility of the spleen.

## Introduction

Sclerosing angiomatoid nodular transformation (SANT), a rare non-neoplastic vascular disease affecting the spleen, is formally known as hemangioma or multinodular hemangioma (1). Officially, SANT was first defined by Martel et al. after reviewing 25 cases from articles published before 2004 (2). It usually occurs in the middle-aged population (3) with a female pre-dominance (4). The vast majority of SANT patients are usually symptom free, with lesions detected occasionally by physical examinations (5) or during the treatment of other unrelated diseases (6). Some cases may present non-specific symptoms, such as abdominal pain (7), nausea, vomiting, and malnutrition (8). To date, the diagnosis of SANT is still a challenge without histological examinations, despite the fact that computerized tomography (CT) and magnetic resonance imaging (MRI) provide imaging support before surgery (5, 6). In clinical practice, open or laparoscopic splenectomy is chosen for the treatment of SANT to avoid the risk of spontaneous rupture (9) and the suspicion of malignancy. In this study, we retrospectively analyzed the clinical and pathological features of five SANT cases admitted to our hospital between July 2010 and November 2019. Our study is a good complement for the whole SANT cohort.

## Materials and Methods

### Ethical Approval

The study protocols were approved by the Ethics Committee of Shaoxing People's Hospital. Written informed consent was obtained from each patient for the publication of this article and any accompanying tables and/or images.

### Patients and Clinical Data

We searched our pathological data and found diagnosed SANT cases based on pathological reports; then these reports were reviewed retrospectively. The clinical data of each patient were collected from the hospital records, including clinical materials and imaging results. Then the data were analyzed retrospectively by qualified staff experienced in the diagnosis of SANT. The post-operative complications were measured with the Clavien-Dindo classification (10).

### Imaging Analysis

Two patients underwent ultrasonography (US) (Mindray, Resona 7 T, 5.0 mHz) in our hospital, and three other patients underwent US (parameters not available) in other hospitals. All the patients underwent a CT scan (GE, BrightSpeed 16/Phillips Brilliance 64) prior to surgery after US which indicated a mass in the spleen. Before examination, a fasting state for 8–10 h was necessary for each patient. The CT scan range was from the diaphragmatic surface to symphysis pubis, and the enhancement consisted of an arterial phase (30–35 s), portal venous phase (60–65 s), and delayed phase (150 s). US results were analyzed by two sonographers, and CT scans were assessed by three radiologists. All the decisions on the diagnosis were made upon consensus.

### Pathological Analysis

All the specimens obtained after surgery were fixed with 4% neutral formalin and processed by dehydration and paraffin embedding. Then the sections (3–4 μm) were subjected to hematoxylin-eosin (HE) staining, followed by observation under a light microscope to confirm the diagnosis of SANT. The immunohistochemical staining was performed by Envision plus. The sections were incubated with primary antibodies including CD34, CD31, CD8, and smooth muscle antibody (SMA) (Maixin Biotech, Fuzhou, China) and then with secondary antibodies plus a DAB chromogenic reagent (Dako). The pathological results were assessed by two pathologists blinded to this study. A detailed discussion was held until a consensus was reached in the presence of any disputes on the pathological reports.

## Results

### Clinical Features

Five SANT cases were found throughout our pathological data, including four cases initially confirmed and one case confirmed after pathological analysis in a retrospective way. Table 1 summarized the clinical data of five SANT patients (male: three; female: two). The mean age of onset was 47.6 years (39–63 years). Three patients presented with mild abdominal pain or discomfort in the left upper quadrant, while the other two patients were asymptomatic. The findings for physical examination were all normal for these patients. All denied a history of smoking, alcohol abuse, hypertension, diabetes, tumors, and other systemic diseases. Laboratory investigations including blood routine, biochemical analysis, and tumor markers were not remarkable in all cases.

**Table 1:** Pre-operative clinical data of SANT cases.

**Case**	**Gender**	**Age of onset**	**Symptoms**	**Signs**
1	Male	39	Incidentally found	Negative
2	Female	63	Mild abdominal pain	Negative
3	Female	40	Mild abdominal discomfort	Negative
4	Male	47	Mild abdominal pain	Negative
5	Male	49	Incidentally found	Negative

### Imaging Findings

**Figure 1:**
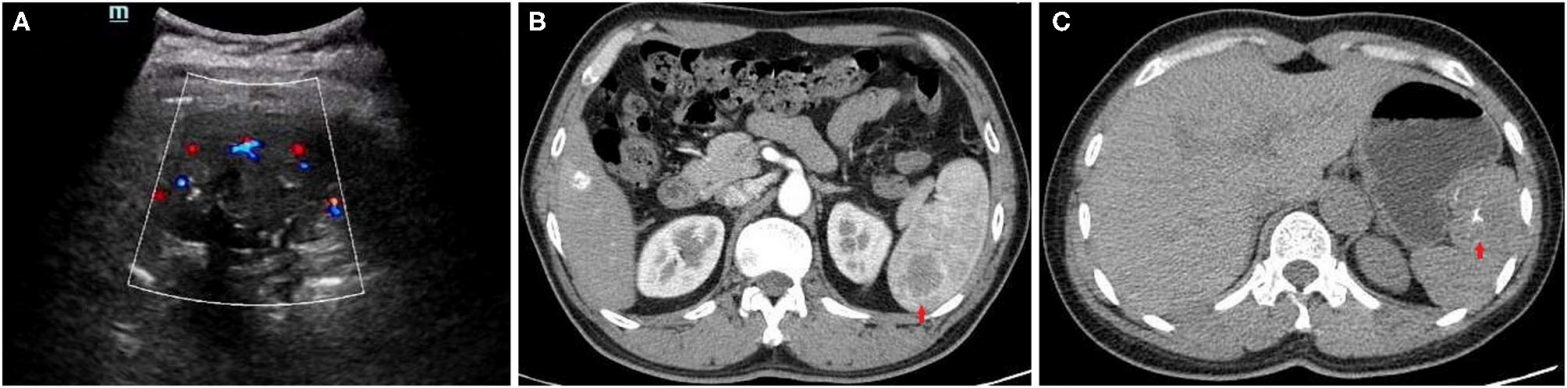
Imaging findings of the SANT patients. **(A)** US showed a heterogeneous echotexture in the spleen, which was featured by internal vascularity on the color Doppler. **(B)** A “spoke wheel” pattern and peripheral enhancement around the SANT lesion was seen during the arterial phase. **(C)** A plain CT scan showed an iso-dense mass with central calcification in the spleen.

Two cases underwent US in our hospital, which showed a hypoechoic or heterogeneous echotexture in the spleen. Meanwhile, color Doppler indicated internal vascularity in the lesion (Figure 1A). The imaging characteristics of the CT scans in the five cases were summarized in Table 2. All the SANT patients presented a solitary hypodense or complicated lesion area on the CT scan that was typically featured by marginal enhancement during the arterial phase (Figure 1B). The lesions were well-circumscribed in a mean size of 3.94 cm (1.6–5.3 cm), which were distributed in the superior pole (*n* = 1), middle pole (*n* = 2), and inferior pole (*n* = 2) of the spleen, respectively. Two cases showed a typical “spoke wheel” pattern (Figure 1B), and calcification in a lesion center was detected in one case (Figure 1C). No patient was diagnosed with SANT in our cohort pre-operatively.

**Table 2:** The imaging characteristics of CT scans in five SANT cases.

**Patients**	**Num**	**Site**	**Size (cm)**	**Enhancement (arterial phase)**	**Spoke wheel**	**Central calcification**
1	1	Inferior pole	5.3 × 5.0	Peripheral	N	N
2	1	Middle pole	1.6 × 1.5	Peripheral	N	N
3	1	Superior pole	5.0 × 4.0	Peripheral	Y	Y
4	1	Inferior pole	3.5 × 2.5	Peripheral	Y	N
5	1	Middle pole	4.3 × 3.9	Peripheral	N	N

*Num, number; cm, centimeter; Y, yes; N, no*.

### Surgical Data

The intraoperative and post-operative data of these five cases are summarized in Table 3. Splenectomy was performed for all patients, with four cases under the laparoscopic approach and one case (spleen size, 13.2 cm × 7.6 cm × 8.4 cm) under the open approach. The median operation time was 151 min (90–200 min), and the intraoperative average blood loss was 160 ml (50–300 ml). After surgery, all the patients showed thrombocythemia with a peak platelet count in a range of 391 × 10^9^/L to 765 × 10^9^/L (Clavien-Dindo classification: Grade II). One patient presented with a Grade A pancreatic fistula (Clavien-Dindo classification: Grade II). These symptoms disappeared after oral administration of aspirin enteric-coated tablets (100 mg/day) for thrombocythemia and intravenous injection of somatostatin (3 mg/day) for the pancreatic fistula. No splenic vein thrombosis or other severe complications were detected. The mean hospital stay after surgery was 10 days (5–21 days). The median follow-up time was 40.5 months (8–116 months) with one patient lost in the follow-up, and no patient showed recurrence during this period.

**Table 3:** Intraoperative and post-operative data of five SANT cases.

**Patients**	**OP mode**	**OP time (min)**	**Blood loss (ml)**	**Peak platelet count (10^**9**^/L)**	**Hospital stay (days)**	**Follow-up (months)**
1	OS	110	150	647	21	116
2	LS	160	300	580	10	Lost
3	LS	90	50	391	5	22
4	LS	200	200	457	7	16
5	LS	195	100	765	7	8

*OP, operation; OS, open splenectomy; LS, laparoscopic splenectomy; ml, milliliter; L, liter*.

### Pathological Characteristics

**Figure 2:**
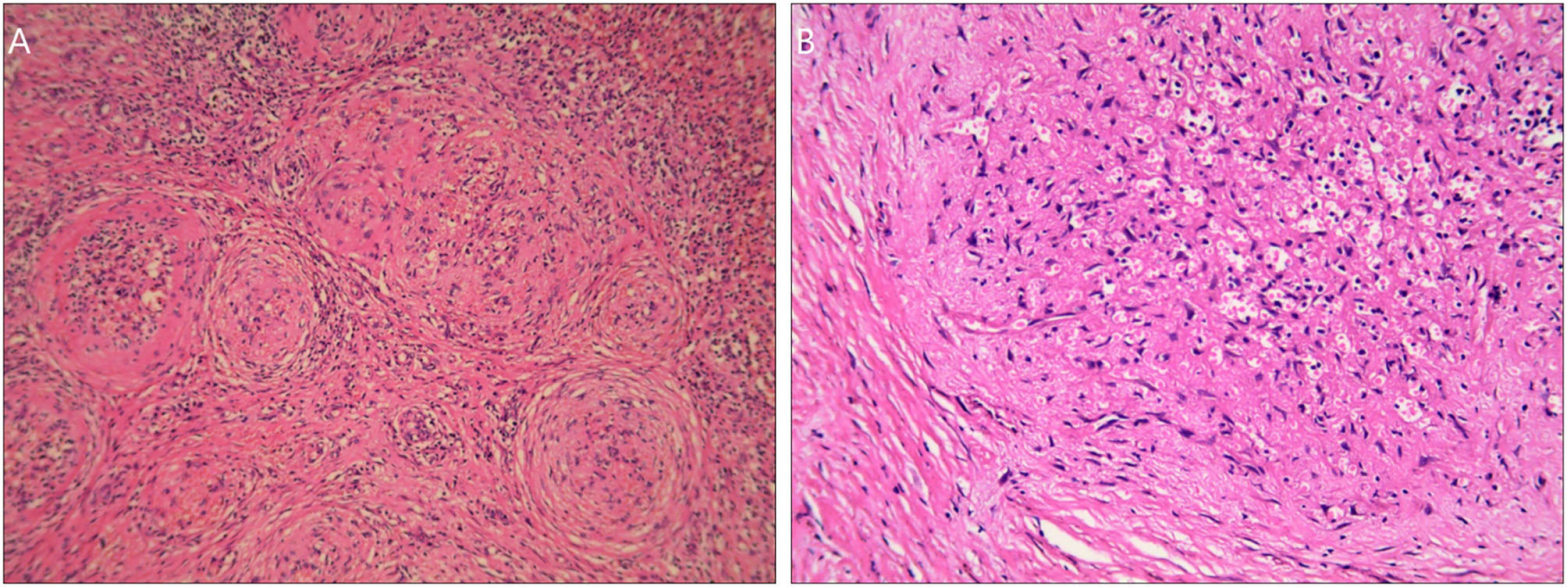
HE staining images of the SANT patients. **(A)** Multiple angiomatous nodules with collagenized fibrous tissues in the peripheral part under a magnification of <200×. **(B)** There was an angiomatous nodule composed of slit-like vessels or sinusoids. Obese endothelial cells lined the vessel lumens. Fusiform and ovoid cells were arranged in a target ring round the nodule under a magnification of 200×.

Grossly, there was a solitary well-circumscribed mass in the splenic parenchyma in each case. The cut surface indicated that there were gray-white stellate scars in the central part, with reddish-brown nodules scattered peripherally. HE staining indicated multiple angiomatous nodules, which were wrapped by collagenized fibrous tissues in all lesions (Figure 2A). The angiomatous nodules were composed of slit-like vessels or sinusoids within which erythrocytes were diffused (Figure 2B). Obese endothelial cells lined the vessel lumens. Fusiform and ovoid cells were arranged in a target ring around the slit-like vessels (Figure 2B). The infiltration of lymphocytes and plasma cells could be seen in the stroma.

**Figure 3:**
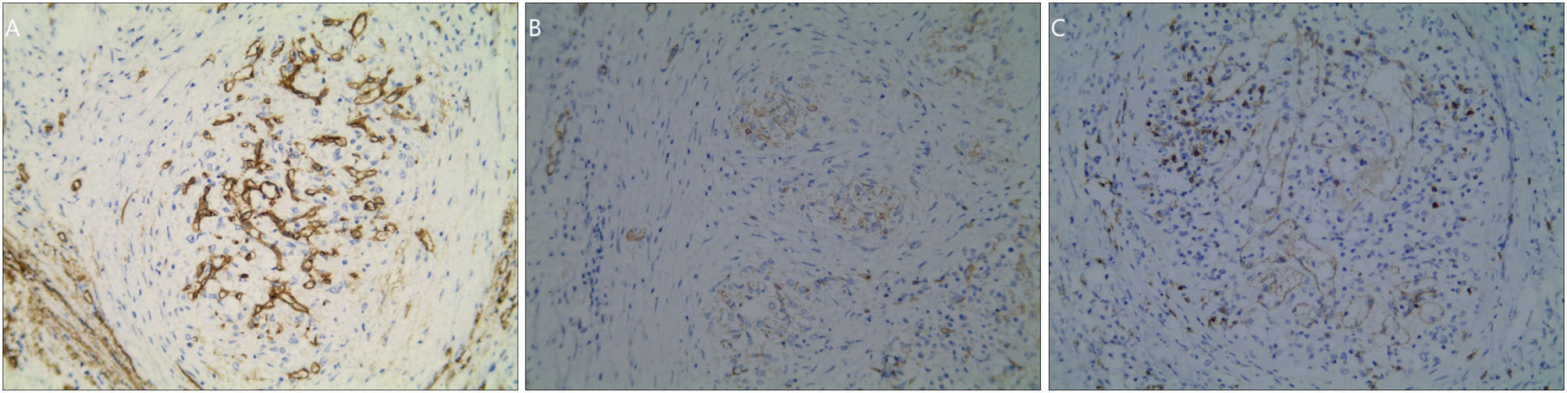
The immunophenotype of the SANT patients under a magnification of 200×. **(A)** Immunohistochemical staining for CD34. **(B)** Immunohistochemical staining for CD31. **(C)** Immunohistochemical staining for CD8.

The immunohistochemical results displayed CD34(−) CD31(+) CD8(+) for sinusoids, CD34(+) CD31(+) CD8(−) for capillaries, CD34(−) CD31(+) CD8(−) for small veins (Figure 3), and SMA(+) for fusiform and ovoid cells.

## Discussion

SANT is a rare benign vascular disease in the red pulp of the spleen. Previously, there was no consensus on the nomenclature for this clinicopathological pattern. It was usually misdiagnosed as hemangioma, hemangiosarcoma, inflammatory pseudotumor, hamartoma (1), and even metastatic tumors (7). In 2004, Martel et al. summarized the pathological features and defined it as SANT for the first time (2). In line with our study, SANT usually affected the middle-aged population with an age of onset between 30 and 60 years (11). There was female pre-dominance for SANT with a female-to-male ratio of 2:1 (4, 12); however, in our study, there was no female pre-dominance (three males and two females), which may be related to the limited sample size. Currently, the etiology of SANT is still unclear. In a previous study, Chang et al. speculated that the pathogenesis of SANT might be associated with vascular insufficiency and the subsequent vascular proliferation for repairing, indicating that SANT was just a polyclonal reactive lesion rather than genuine neoplasm (13). To our best knowledge, it is still a great challenge to carry out epidemiological studies for SANT as it shows a low morbidity.

The clinical manifestation of SANT is asymptomatic or non-specific, with most lesions identified incidentally (5) or during the treatment of other unrelated diseases (6). Others exhibited featureless symptoms such as abdominal pain (7), nausea, vomiting, and malnutrition (8). Recently, Pelizzo et al. reported a case of SANT with spontaneous mass rupture and intraperitoneal hemorrhage in a 9 week-old female infant (9). In our study, two patients were detected incidentally, and the other three had slight abdominal pain or discomfort, which was consistent with most SANT cases.

Given a lack of specific biomarkers for SANT, pre-operative imaging studies should be of relatively great importance. Although US can conveniently detect the heterogeneous echotexture in the spleen and the internal vascularity on color Doppler can provide some clues for SANT, definite diagnosis is still a challenge due to the various appearances of SANT in US images (14). In the present study, two patients underwent Doppler scans in our hospital, which indicated only an euangiotic mass in the spleen. For the CT scan, SANT usually presented a hypo- or iso-dense complex mass which was located in any site of the spleen just like in our cases (5). According to the literature, central calcification and a “spoke wheel” pattern were usually considered typical manifestations in CT images, but these signs were not available in all patients (5, 6, 15). For instance, of the five cases in our study, only one case had central calcification, and two cases showed a “spoke wheel” pattern. However, all five patients presented peripheral enhancement around the SANT lesion during the arterial phase. Thus, this imaging phenomenon should be taken into consideration for the diagnosis of SANT based on CT scans. MRI had some advantages in displaying certain imaging features of SANT (6). As there were no patients who underwent this examination in our study, we could not hold a deep discussion about it.

Generally, splenectomy is considered as the first-line option for SANT (12), avoiding the risk of spontaneous rupture (9) and the suspicion of malignancy. Open and laparoscopic approaches are alternative options depending on the different situations of the patients (16). For patients with a large spleen after a pre-operative imaging assessment, such as the open case in this study, an open surgery is preferred as the large spleen may limit the laparoscopic space and may increase difficulties during the laparoscopic approach. However, for surgeons experienced in laparoscopy, a laparoscopic surgery can still be tried. In our study, there were no significant differences in the operation time, blood loss, and post-operative complications between patients treated by different surgical approaches. However, the hospital stay seemed to be shorter for patients who underwent the laparoscopic approach. These results should be further verified due to the limited number of cases. During the laparoscopic approach, there is a high possibility of splenic rupture. Nowadays, confusion still arises in the pre-operative diagnosis of SANT although it is a benign vascular lesion. Furthermore, we could not absolutely exclude the probability of malignancy. Thus, we propose that the preservation of spleen integrality is of great importance during surgery, in order to achieve en bloc resections. To our best knowledge, the prognosis of SANT is favorable after surgical resections (6, 17) with no recurrence in previous literature.

To date, the pathological characteristics of SANT have been well-defined. Macroscopically, SANT usually presented as an isolated well-demarcated mass with a gray-white stellate scar in the central part. Besides, reddish-brown nodules were localized in the peripheral sites (2, 6). Multiple angiomatous nodules wrapped by collagenized fibrous tissues were the most typical traits for SANT microscopically. The nodules were usually composed of slit-like vessels or sinusoids and fibrous tissues constituted of fusiform and ovoid cells which were arranged in target ring around the nodules (2, 6, 11). Erythrocytes could be seen in the vessel lumens, while the stroma was infiltrated by lymphocytes and plasma cells. All these features were well in line with our cases.

According to previous literature, the immunophenotype of SANT was almost similar among reported cases, which was featured by CD34(−) CD31(+) CD8(+) for sinusoids, CD34(+) CD31(+) CD8(−) for capillaries, and CD34(−) CD31(+) CD8(−) for small veins (2, 18, 19). These three types of vessels were seemingly mimicking the structure of red pulp, which indicated that SANT may originate from red pulp.

## Conclusions

SANT is a rare benign vascular entity affecting the spleen. A CT scan is relatively important for SANT diagnosis pre-operatively. Peripheral enhancement around the lesion during the arterial phase should be taken into consideration as an imaging sign on CT scans for SANT diagnosis. Open or laparoscopic splenectomy is an alternative for SANT treatment. Preservation of spleen integrality should be preferred, especially during the laparoscopic approach, because of the probability of malignancy and the fragility of the spleen. According to pathological features, SANT may originate from the red pulp of the spleen.

## Data Availability Statement

The raw data supporting the conclusions of this article will be made available by the authors, without undue reservation.

## Ethics Statement

The studies involving human participants were reviewed and approved by The Ethics Committee of Shaoxing People's Hospital. The patients/participants provided their written informed consent to participate in this study. Written informed consent was obtained from the individual(s) for the publication of any potentially identifiable images or data included in this article.

## Author Contributions

HS wrote the manuscript. FL revised the manuscript. BL completed the data analysis. ZS collected the data. All authors have read and approved the article.

## Conflict of Interest

The authors declare that the research was conducted in the absence of any commercial or financial relationships that could be construed as a potential conflict of interest.

## Abbreviations

SANT: sclerosing angiomatoid nodular transformation
CT: computerized tomography
US: ultrasonography.
